# Thin-Film Coated Plastic Wrap for Food Packaging

**DOI:** 10.3390/ma10070821

**Published:** 2017-07-18

**Authors:** Hsin-Yu Wu, Ting-Xuan Liu, Chia-Hsun Hsu, Yun-Shao Cho, Zhi-Jia Xu, Shu-Chuan Liao, Bo-Han Zeng, Yeu-Long Jiang, Shui-Yang Lien

**Affiliations:** 1 Graduate Institute of Optoelectronic Engineering and Department of Electrical Engineering, National Chung Hsing University, Taichung 40227, Taiwan; xinmarchentic@gmail.com (H.-Y.W.); yljiang@nchu.edu.tw (Y.-L.J.); 2 Department of Electrical Engineering, Da-Yeh University, Chunghua 51591, Taiwan; f0003209@gmail.com; 3 Department of Materials Science and Engineering, Da-Yeh University, Chunghua 51591, Taiwan; cstcaptive@gmail.com (C.-H.H.); ysc@mail.dyu.edu.tw (Y.-S.C.); s313326229@gmail.com (Z.-J.X.); 4 Bachelor Program for Design and Materials for Medical Equipment and Devices, Da-Yeh University, Changhua 51591, Taiwan; liaozizi@mail.dyu.edu.tw (S.-C.L.); z0978588769@gmail.com (B.-H.Z.); 5 Institute of Biomedical Engineering, National Taiwan University, Taipei 106, Taiwan

**Keywords:** polymethylpentene, food package, inductively coupled plasma chemical vapor deposition, water vapor transmission rate, shelf-life

## Abstract

In this study, the antimicrobial property and food package capability of polymethylpentene (PMP) substrate with silicon oxdie (SiO*_x_*) and organic silicon (SiC*_x_*H*_y_*) stacked layers deposited by an inductively coupled plasma chemical vapor deposition system were investigated. The experimental results show that the stacked pair number of SiO*_x_*/SiC*_x_*H*_y_* on PMP is limited to three pairs, beyond which the films will crack and cause package failure. The three-pair SiO*_x_*/SiC*_x_*H*_y_* on PMP shows a low water vapor transmission rate of 0.57 g/m^2^/day and a high water contact angle of 102°. Three-pair thin-film coated PMP demonstrates no microbe adhesion and exhibits antibacterial properties within 24 h. Food shelf life testing performed at 28 °C and 80% humidity reports that the three-pair thin-film coated PMP can enhance the food shelf-life to 120 h. The results indicate that the silicon-based thin film may be a promising material for antibacterial food packaging applications to extend the shelf-life of food products.

## 1. Introduction

There has been growing interest in recent years to develop materials with antibacterial properties which improve food safety and shelf-life. Antibacterial packaging is one of the most promising active packaging systems [1,2,3,4,5]. In addition to the basic properties of packaging materials, antimicrobial food package should prevent the gain of moisture, hinder microbial contamination, and act as a barrier against the permeation of water vapor, oxygen, carbon dioxide, and other volatile compounds.

Basic packaging materials, such as paper and paperboard, plastic, glass, and metal, are used to fulfill the capabilities and requirements of packaged foods, depending on their type. However, there has been an ever-increasing endeavor to develop different kinds of food packaging materials in order to extend the shelf-life with improved water vapor resistivity and antibacterial property. Silicon oxide (SiO*_x_*) barrier films with high density and mechanical resistance, as well as a low water permeation rate can be deposited using plasma-enhanced chemical vapor deposition at low temperatures [6,7]. It has been proven that SiO*_x_* is useful in improving the gas barrier properties of some food packaging materials such as polyethylene terephthalate [8,9] and polyvinyl chloride [10]. Polymethylpentene (PMP) is a thermoplastic polyolefin with excellent mechanical and thermal stabilities [11]. However, PMP plastic food wraps have a high moisture permeation rate and there are few research studies related to the improvement of the water vapor resistivity and antibacterial property of PMP.

In this paper, inorganic SiO*_x_* and organic silicon (SiC*_x_*H*_y_*) stacked layers were prepared on a PMP substrate by using inductively coupled plasma chemical vapor deposition (ICPCVD). Silicon oxide-based thin film is widely known as a great barrier against water vapor [12], but SiO*_x_* may likely have cracks induced by the accumulation of internal residual compressive stress when the film thickness increases. SiC*_x_*H*_y_* films with good tensile stress were thus stacked with SiO*_x_* to reduce the total internal stress. The stacked layers were continually deposited in the same chamber without breaking the vacuum conditions. The pair of the SiO*_x_*/SiC*_x_*H*_y_* stacked layers was varied in order to reduce the water vapor transmission rate (WVTR). Finally, the food package capability of PMP with SiO*_x_*/SiC*_x_*H*_y_* stacked layers was evaluated, and the PMP with the stacked layers demonstrated greatly improved antibacterial properties and extended the food shelf-life.

## 2. Materials and Methods

### 2.1. Materials

Silicon-based thin films, such as SiO*_x_* and SiC*_x_*H*_y_* layers, were prepared on PMP substrates by a 13.56-MHz radio frequency ICPCVD system. A gas mixture of oxygen (O_2_) and tetramethylsilane (TMS) was used to deposit SiO*_x_*, while argon (Ar) and TMS were used to deposit SiC*_x_*H*_y_*. For SiO*_x_* layers, the gas flow ratio of O_2_ to TMS was 4. For SiC*_x_*H*_y_* layers, the gas flow ratio of Ar to TMS was 0.03. The radio frequency power for SiO*_x_* and SiC*_x_*H*_y_* were 600 and 400 W, respectively. The deposition pressure was 5 mTorr and the temperature was kept at 90 °C. In our previous study, a minimal internal stress was obtained when the thicknesses of SiO*_x_* and SiC*_x_*H*_y_* were 300 and 30 nm, respectively. The pair number of the silicon-based stacked layers was varied from one to three.

### 2.2. Antibacterial Efficacy Test

The bacterial strains used in this study were *Escherichia coli* (*E. coli*) (ATCC, strain 25922) from Creative Life Science Co., Ltd. (Taipei, Taiwan). The antimicrobial effect was tested by the Kirby-Bauer diffusimetrical method. The bacteria was incubated for 17 h with vigorous shaking (250 rad/min) at 37 °C, and were obtained from a bacterial inoculum, which was standardized according to the McFarland scale, yielding to 10^7^ CFU/mL. Luria-Bertani (LB) medium was inoculated with that inoculum and afterwards, different pairs of the SiO*_x_*/SiC*_x_*H*_y_* films with a diameter of 1 cm were applied on the surface of the medium. The antimicrobial property against the strains of *E. coli* was assessed by measuring the ratio of microbes to sample area after 0, 2, 4, 8, 12, 24, 48, and 72 h of incubation at 37 °C.

### 2.3. Bread Packaging Test

Four fresh toast slices (size: 10 cm × 5 cm) without preservatives were packed in the PMP without and with different pairs of SiO*_x_*/SiC*_x_*H*_y_* films. The toast slices were stored at a relatively humidity of 80% and a temperature of 28 °C. Photographs of the toast slices were taken after 120 h, and ImageJ computing software (National Institute of Mental Health, Maryland, MD, USA) [13,14] was used to evaluate the area of fungal contamination.

### 2.4. Characterization

The topographical morphologies of the SiO*_x_*/SiC*_x_*H*_y_* films were observed by optical microscopy (OM, MICROTECH MX810-RF, M&T Optics, Co., Ltd., Taipei, Taiwan). The WVTR of the films was measured by a WVTR permeation instrument (PERMATRAN-WR Model 3/61, Mocon Inc., Minneapolis, MN, USA) under the conditions of 40 °C and 100% relative humidity (RH) [15,16]. Water contact angle measurements were performed with a pocket goniometer (Model PGX, Testing Machines Inc., Veenendaal, The Netherlands) using 2 μL droplets, with at least five repetitions for each sample. The morphologies of *E. coli* grown on PMP without and with different pairs of SiO*_x_*/SiC*_x_*H*_y_* films were observed using high resolution thermal field emission scanning electron microscopy (HRFEG-SEM, JSM-7610F, JEOL, Tokyo, Japan) at 10 kV.

## 3. Results and Discussion

### 3.1. Properties of Silicon-Based Thin Films

**Figure 1:**
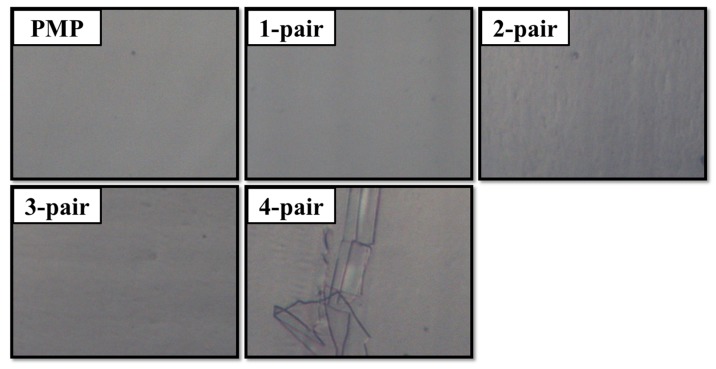
Optical microscope images of the polymethylpentene (PMP) without, with one-, two-, three- and four-pair SiO*_x_*/SiC*_x_*H*_y_* films.

The OM images of PMP with different pairs of SiO*_x_*/SiC*_x_*H*_y_* films are shown in Figure 1. The PMP without, with one-, two-, and three-pair SiO*_x_*/SiC*_x_*H*_y_* stacked layers show smooth surface and good structural integrity without significant pores or cracks. The four-pair SiO*_x_*/SiC*_x_*H*_y_* stacked layer exhibits crack patterns on the film surface. One possible reason for the cracks might be attributed to the heat accumulation on the substrate during the deposition process. PMP substrate is very likely to curl under long plasma deposition as the ion bombardment will heat the PMP substrate. Another reason might be due to the increased internal residual stress as the pair number increases. Overall, the maximal pair number of SiO*_x_*/SiC*_x_*H*_y_* on PMP was limited to three in order to avoid film cracking and packaging failure.

**Figure 2:**
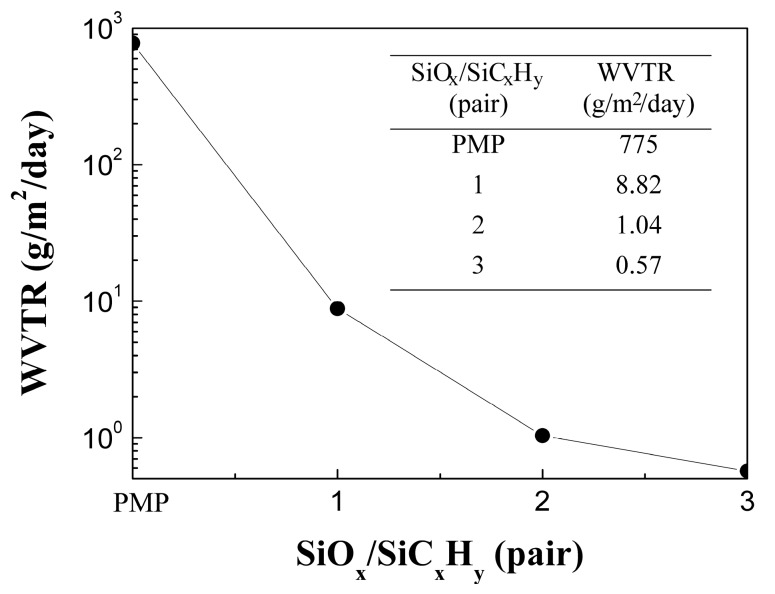
Water vapor transmission rate for the PMP without and with one-, two-, and three-pair SiO*_x_*/SiC*_x_*H*_y_* layers at 40 °C/100% relative humidity (RH).

Figure 2 shows the WVTR at 40 °C/100% RH of the SiO*_x_*/SiC*_x_*H*_y_* stacked layers with one, two, and three pairs. It can be seen that the WVTR of the PMP substrate is as high as 775 g/m^2^/day. The one-pair SiO*_x_*/SiC*_x_*H*_y_* significantly reduced WVTR values to 8.82 g/m^2^/day, which further decreases to 1.04 g/m^2^/day for the two-pair SiO*_x_*/SiC*_x_*H*_y_*. The three-pair SiO*_x_*/SiC*_x_*H*_y_* demonstrates a WVTR value of 0.57 g/m^2^/day. The reduction in WVTR can be related to the increase of the total thickness of the gas barrier, which causes the water vapor to spend more time traveling through the barrier layer to the other side. A WVTR value ranging from 0.01 to 1 g/m^2^/day is suggested for food packaging [17]. The PMP substrate can satisfy this requirement if the three-pair SiO*_x_*/SiC*_x_*H*_y_* film is coated.

**Figure 3:**
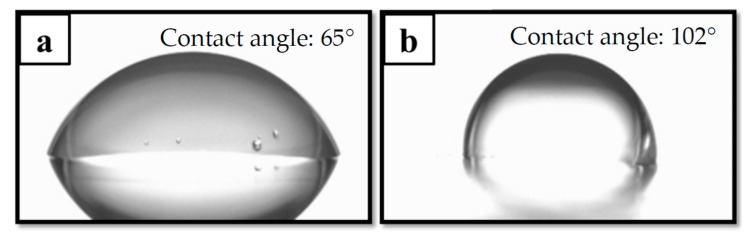
Water contact angle images for the PMP (**a**) without and (**b**) with SiC*_x_*H*_y_*/three-pair SiO*_x_*/SiC*_x_*H*_y_* stack.

The hydrophobicity of a packaging material is a major concern, as it limits water vapor attachments. However, the SiO*_x_*/SiC*_x_*H*_y_* stacked films may exhibit a hydrophilic surface with the water contact angle <90°, due to the hydrophilicity of the silicon oxide nature. In this study, a 30 nm SiC*_x_*H*_y_* layer was intentionally added to the top of the stacked structure to produce a hydrophobic surface. The water contact angle measurement results for the PMP without, and with SiC*_x_*H*_y_*/three-pair SiO*_x_*/SiC*_x_*H*_y_* stack are shown in Figure 3. The water contact angles for the PMP without and with the stacked layer are approximately 65° and 102°, respectively. Hydrophobic properties are known to be observed at contact angles above 90° [18]. The PMP with the stacked layer is confirmed to attain a desirable level of hydrophobicity.

### 3.2. Evaluation of the Antibacterial Effect

**Figure 4:**
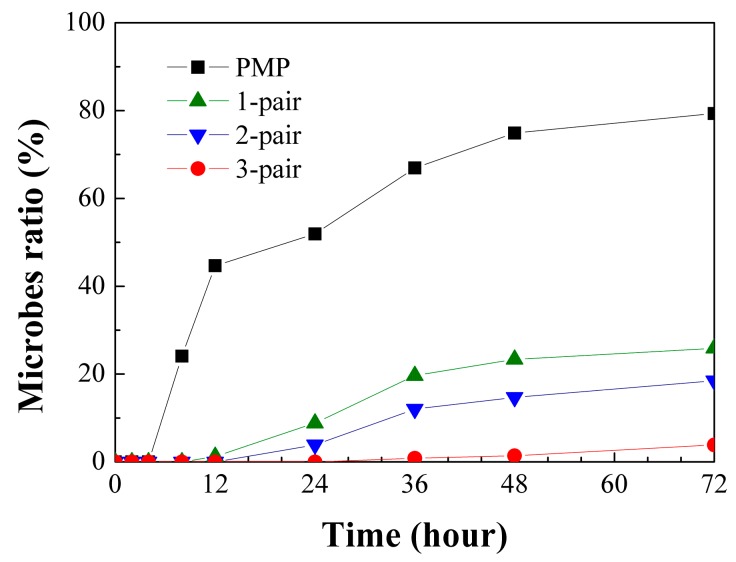
Ratio of microbe adhesion area to sample area for PMP without and with different pairs of SiO*_x_*/SiC*_x_*H*_y_* stacked films as a function of time.

**Figure 5:**
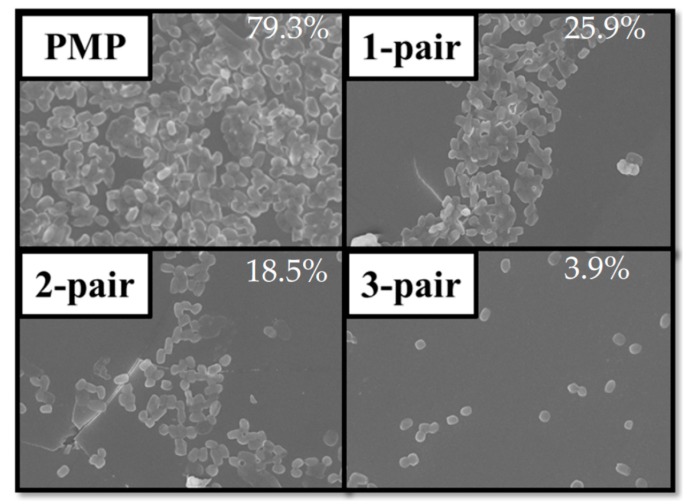
SEM images for *Escherichia coli* adhesion on PMP without and with different pairs of SiO*_x_*/SiC*_x_*H*_y_* stacked films at 72 h. The percentage represents the ratio of microbes to total area.

The adhesion of the microbes covered by PMP without and with stacked layers is observed by SEM, and the ratio of microbes to sample area as a function of time is shown in Figure 4. For the PMP substrate without the stacked layers, the microbe ratio is negligible for the first 8 h. Afterwards, the ratio rapidly increases to 80% at 72 h. With the stacked layers, the microbe ratio significantly decreases as compared to that of the PMP without stacked layers. The one- and two-pair stacked layers can lead to a microbe ratio of 0% within 12 h, while the PMP with the three-pair stacked layer demonstrates no microbe adhesion within 24 h. This result indicates the great antibacterial function of the PMP with the SiO*_x_*/SiC*_x_*H*_y_* stacks. The SEM images at a certain position on each sample at 72 h are shown in Figure 5. The microbe ratios are 79.3%, 25.9%, 18.5%, and 3.9% for the PMP without, and with one-, two-, and three-pair stacked layers, respectively.

**Figure 6:**
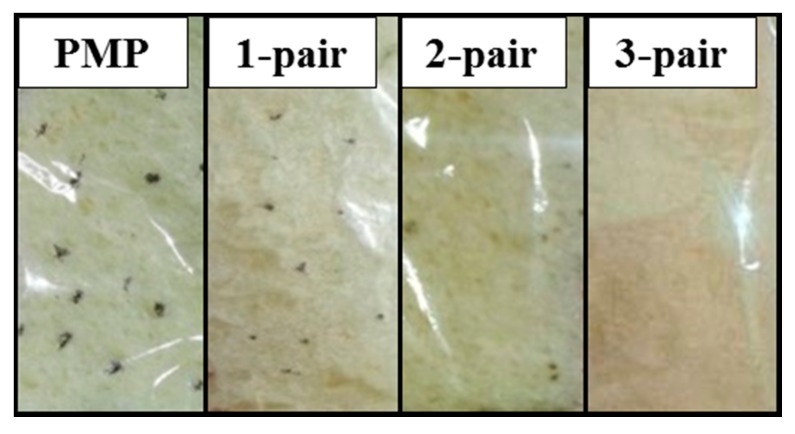
Images of food packed by PMP without and with different pairs of SiO*_x_*/SiC*_x_*H*_y_* stacked film.

The PMP substrates without and with different pairs of SiO*_x_*/SiC*_x_*H*_y_* stacked films are applied to food packaging, and the appearances of the packed toasts stored at 80% RH and 28 °C after 120 h are shown in Figure 6. In this study, we choose the toast without any added preservatives. It can be observed that fungi grow on the toasts packed by the PMP without, as well as with one-pair and two-pair SiO*_x_*/SiC*_x_*H*_y_* stacked films. While the toast packed with the three-pair stacked film shows the absence of fungi growth. The extent of the fungi growth is evaluated as listed in Table 1. The area ratio of the fungi to sample surface is greater than 2% for the PMP substrate without the stacked films. The ratio is significantly reduced to less than 1% for the PMP with one pair of stacked film. The two-pair stacked film further decreases the ratio to 0.7%. Finally, no observable fungi are presented for the three-pair stacked film. The trend of this result can be directly linked to the WVTR values. Lower WVTR value means decreased water and vapor pass through the packaging layer, and this reduces fungi growth.

**Figure 7:**
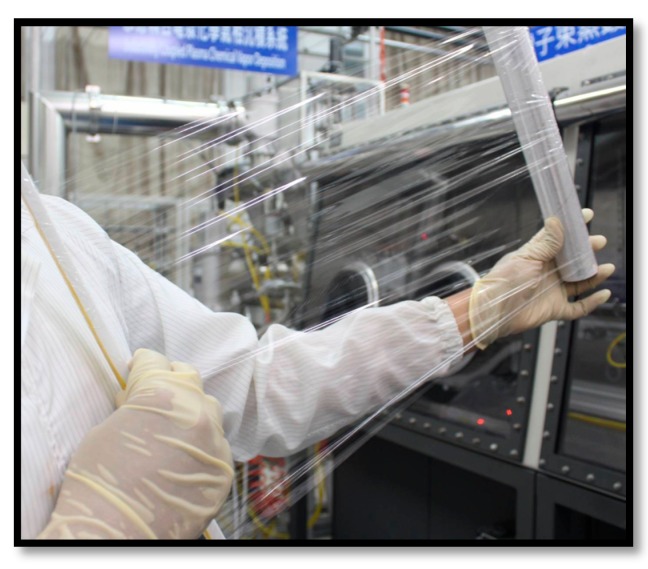
Photograph of stretched and rolled PMP with three-pair SiO*_x_*/SiC*_x_*H*_y_* stack.

The package technique in this work can be used in large area (25 cm × 25 cm) or roll-to-roll deposition, as shown in Figure 7. The PMP with the three-pair stacked film can easily be stretched and rolled without obvious film cracking, and the film retains its flexibility. The SiO*_x_*/SiC*_x_*H*_y_* stacked film demonstrates great potential to be used in food package applications.

## 4. Conclusions

Silicon-based SiO*_x_* and SiC*_x_*H*_y_* layers are prepared on PMP using the ICPCVD system. The PMP with the three-pair SiO*_x_*/SiC*_x_*H*_y_* stack shows a low WVTR value of 0.57 g/m^2^/day and a high contact angle value of 102°. The three-pair stack exhibits a remarkable antibacterial ability against *E. coli* within 24 h. The food package testing shows that the PMP with the stacked films can extend the food shelf-life to 120 h at 28 °C and 80% RH. These results suggest that the SiO*_x_*/SiC*_x_*H*_y_* stack has great moisture barrier property as well as antimicrobial characteristics, making it useful in food packaging applications.

## Figures and Tables

**Table 1:** Extent of fungi growth area ratio for food packed by PMP without and with different pairs of SiO*_x_*/SiC*_x_*H*_y_* stacked film.

Type of Packaging	PMP	1-Pair	2-Pair	3-Pair
Fungi growth ^1^	+++	++	+	−

^1^ absence of fungal growth, + fungal growth on <0.7% of the surface, ++ fungal growth on <1% of the surface, +++ fungal growth on >2% of the surface.
